# Setting the Stage for Notch: The *Drosophila* Su(H)-Hairless Repressor Complex

**DOI:** 10.1371/journal.pbio.1002524

**Published:** 2016-07-26

**Authors:** Tilman Borggrefe, Franz Oswald

**Affiliations:** 1 Institute of Biochemistry, University of Giessen, Giessen, Germany; 2 Center for Internal Medicine, Department of Internal Medicine I, University Medical Center Ulm, Ulm, Germany

## Abstract

Notch signaling is iteratively used throughout development to maintain stem cell potential or in other instances allow differentiation. The central transcription factor in Notch signaling is CBF-1/RBP-J, Su(H), Lag-1 (CSL)—Su(H) in *Drosophila*—which functions as a molecular switch between transcriptional activation and repression. Su(H) represses transcription by forming a complex with the corepressor Hairless (H). The Su(H)-repressor complex not only competes with the Notch intracellular domain (NICD) but also configures the local chromatin landscape. In this issue, Yuan and colleagues determined the structure of the Su(H)/H complex, showing that a major conformational change within Su(H) explains why the binding of NICD and H is mutually exclusive.

## Abstract 2

This Primer examines recent research that reveals the structural rearrangements that determine whether the Notch pathway transcription factor CSL/Su(H) activates or represses transcription.

Notch signaling is one of only a handful of highly conserved signal transduction pathways that translate extracellular cues into changes in gene expression. Upon ligand binding, the Notch receptor is proteolytically cleaved, resulting in the release of the Notch intracellular domain (NICD). NICD subsequently migrates into the nucleus and binds to the transcription factor CBF-1/RBP-J, Su(H), Lag-1 (CSL), which in *Drosophila melanogaster* is known as Suppressor of Hairless, or Su(H) (see also Fig 1). The activation of Notch target genes requires the recruitment of the coactivator complex, which is composed of transcription cofactors like mastermind (MAM) and the histone acetyltransferase (HAT) p300 [1,2]. Distinct positive histone marks, like H3K27ac and H3K4me3, characterize this transcriptionally active state (see Fig 1 [right side: “ON”]). In the absence of a Notch signal, CSL recruits a corepressor complex containing histone deacetylases (HDACs) and H3K4 demethylases (KDMs) (see Fig 1 [left side: “OFF”]; reviewed in [3]). Thus, the transcription factor CSL functions as a molecular switch by binding either to corepressors or coactivators. In this issue, Yuan et al. [4] unveil the structural and molecular details of the Su(H) corepressor complex in *Drosophila*. This is not only important for the Notch community but is also a pioneering example of how other central transcriptional switches in other evolutionary conserved signal transduction pathways may work. Interestingly, it is known that epigenetic modifiers such as HDACs and HATs as well as histone lysine demethylases (KDMs) and histone lysine methyltransferases (KMTs) contribute to the fine-tuning of the transcriptional switch by dynamically regulating the chromatin environment at the core promoters and/or at enhancers. At the heart of this, there is a single transcription factor—or most likely an ensemble of several transcription factors—orchestrating the signaling output even before the ligand binds to its cognate receptor. Thus, understanding the molecular details of the key players in such switches—for example, the transcription factor Su(H) bound to its cofactor Hairless—is the eye-opener for designing mutants that allow for differentiation between activating and repressing mechanisms by changing individual amino acids. As an excellent example, this has been done in the study by Yuan et al. using the power of crystallography together with *Drosophila* genetics, which is particularly well-studied in regard to Notch signaling.

**Fig 1:**
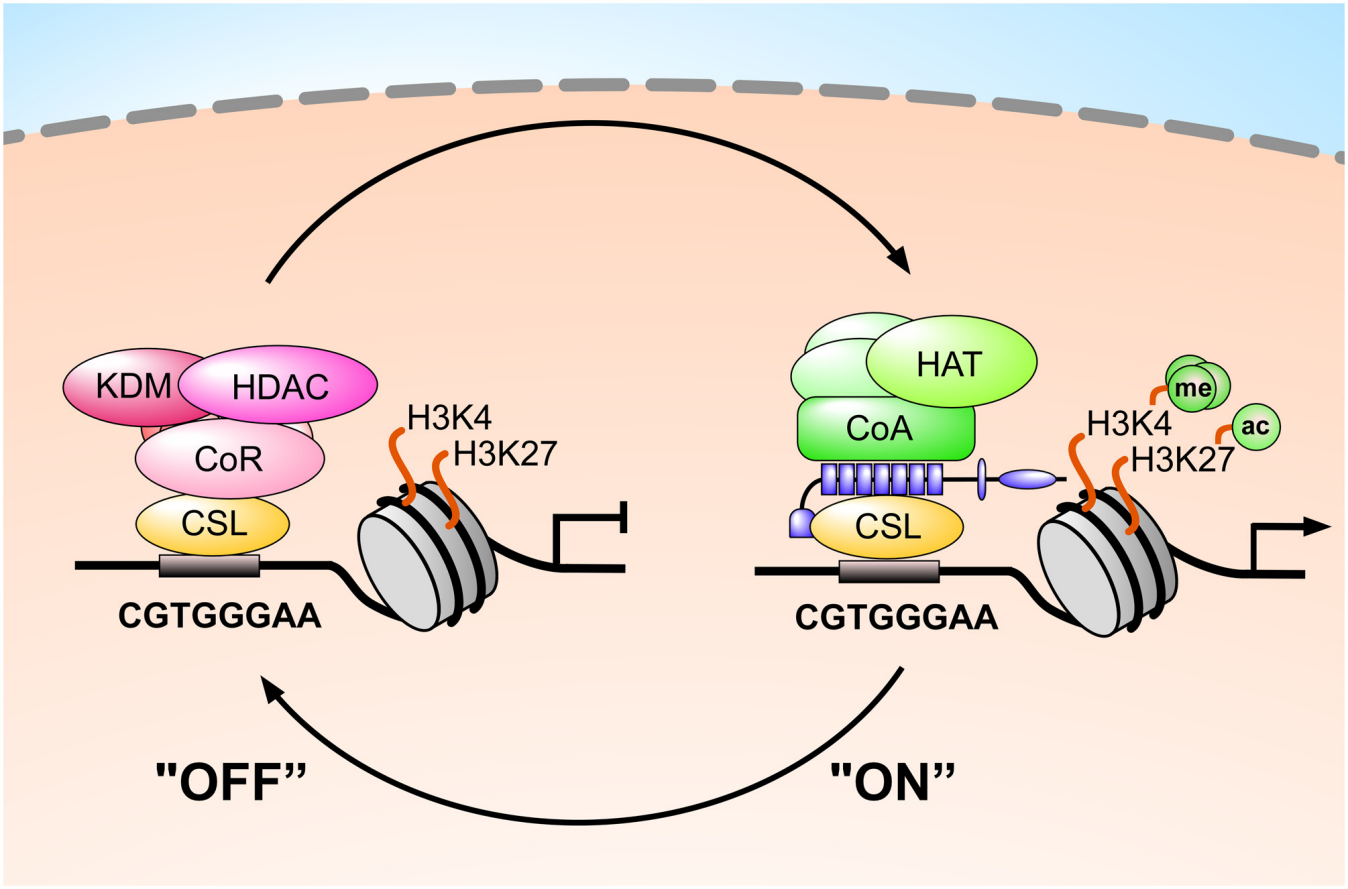
CSL functions as a molecular switch by binding either to corepressors or coactivators: Without an active Notch signal (“OFF”), CSL recruits corepressor complexes. H3K4 Demethylase (KDM) and HDAC activity erase the active chromatin marks H3K4me3 and H3K27ac, establishing a repressed chromatin state (left). After nuclear translocation, NICD (blue) interacts with CSL, recruiting a coactivator complex (right). H3K4-methyltransferase and histone acetyltransferase (HAT) activity establish the active chromatin marks H3K4me3 and H3K27ac, setting the chromatin in a transcriptional active state (“ON”).

## The Notch Transcriptional Activator Complex

**Fig 2:**
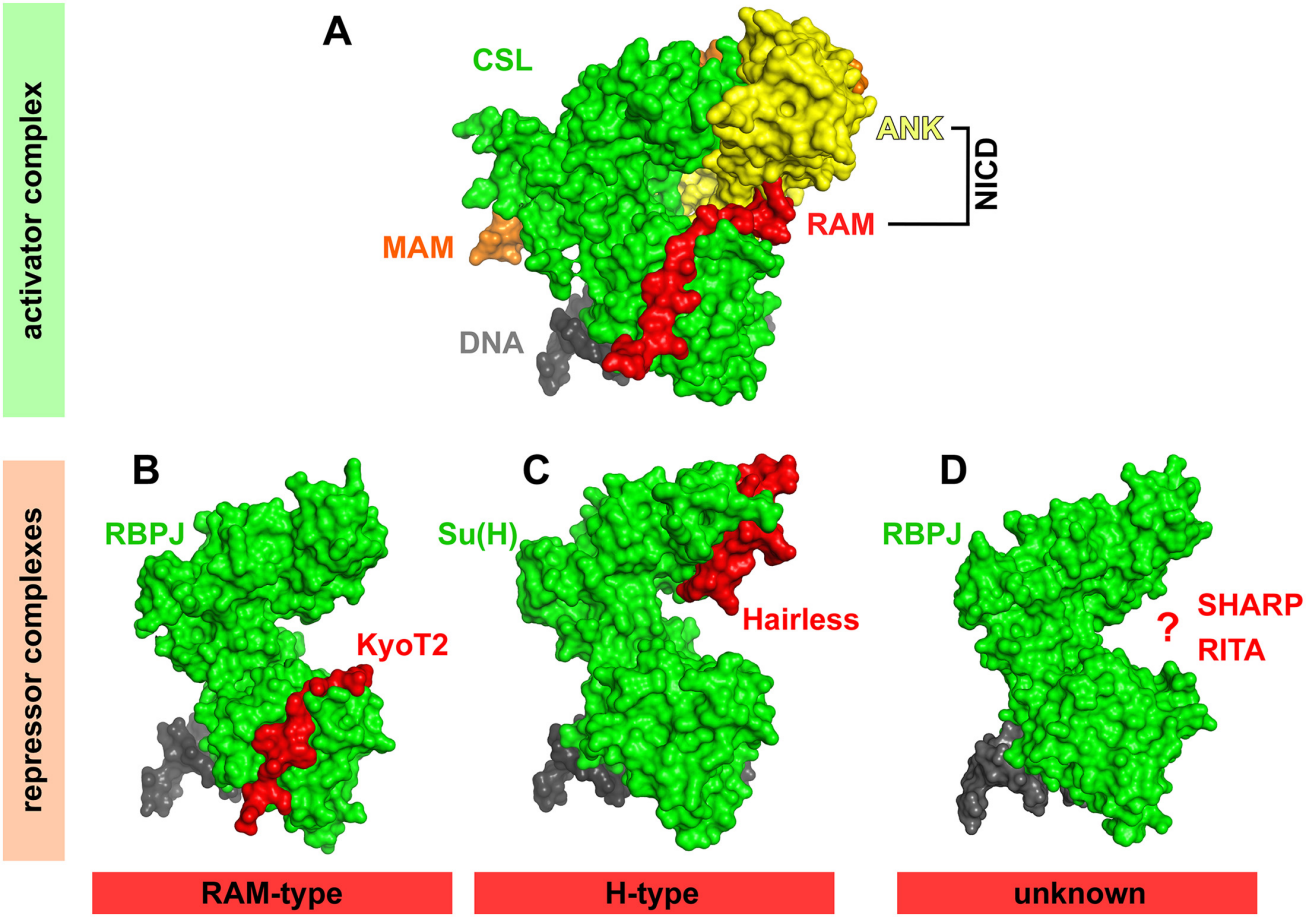
Surface views of the CSL coactivator complex (upper) and corepressor complexes (lower). (A) The DNA-bound CSL activator complex consists of CSL (green), NICD (RAM domain, red; ankyrin repeats, yellow), and mastermind (MAM, orange). (PDB-ID: 1TTU). (B) KyoT2 (red) interacts with the BTD of CSL, similar to the NICD RAM domain (RAM-type). (PDB-ID: 4J2X). (C) Hairless interacts with the CTD of Su(H), resulting in a dramatic change of CTD conformation (H-type). (PDB-ID: 5E24). (D) The crystal structure of the SMRT/HDAC1 associated repressor protein (SHARP)-CSL corepressor complex and the CSL-RBPJ interacting and tubulin associated (RITA) corepressor complex is unknown at the moment (PDB-ID, RBPJ bound to DNA: 3BRG).

In order to understand molecular mechanisms of biological processes, crystal structures are extremely insightful. Table 1 summarizes the structures that contain CSL-mediated transcription complexes. Historically, the mammalian transcription factor CSL (also known as recognition binding protein of Jκ [RBP-J or RBP-Jκ]) was discovered by Honjo and colleagues in the 1990s [5] and later was revealed to be the mammalian ortholog of Su(H) from *Drosophila* [6]. The original CSL-DNA complex structure showed that CSL is a distant relative of the Rel homology–containing transcription factor family [7]. The structure clearly shows that CSL is composed of three domains: N-terminal domain (NTD), β-trefoil domain (BTD), and C-terminal domain (CTD) [7–9]. The NTD and BTD domains are involved in DNA binding. Subsequently, two landmark studies determined the structure of the Notch activator complex, comprising CSL, a small N-terminal peptide of MAM, and the RAM (RBP-J associated molecule) and ANK (ankyrin repeats) domains of NICD. Importantly, as depicted in Fig 2A and described in [10,11], the RAM domain of NICD interacts with the BTD of CSL, whereas MAM is sandwiched between the surface formed between the CTD and ANK. This macromolecular assembly is supported by experiments demonstrating that the ~70 amino acids of MAM seen in the structure are sufficient to block transcription of Notch target genes in a dominant-negative manner [12].

**Table 1:** Available CSL complex structure data (protein data bank [PDB] database).

PDB-ID	Complex	Species	Reference
1TTU	CSL bound to DNA	*Caenorhabditis elegans*	[7]
2FO1	activator complex bound to DNA*	*C*. *elegans*	[10]
2F8X	activator complex bound to DNA^#^	*Homo sapiens*	[11]
3BRD	CSL-RAM bound to DNA	*C*. *elegans*	[8]
3BRF	CSL-RAM bound to DNA	*C*. *elegans*	[8]
3BRG	CSL bound to DNA	*Mus musculus*	[8]
3NBN	activator complex dimer bound to DNA	*H*. *sapiens*	[13]
3V79	activator complex bound to DNA*	*H*. *sapiens*	[14]
3IAG	CSL bound to DNA	*M*. *musculus*	[9]
4J2X	repressor complex bound to DNA**	*M*. *musculus*, *H*. *sapiens*	[15]
5E24	repressor complex bound to DNA***	*Drosophila melanogaster*	[4]

*(CSL/ANK/RAM/MAM),

^#^(CSL/ANK/MAM),

**(CSL/KyoT2),

***(Su[H]/H).

## The CSL [Su(H)] Transcriptional Repressor Complex

Like the Notch locus itself, the Hairless (H) locus was discovered in 1923 by Bridges and Morgan as a haploinsufficient mutation in *Drosophila* (reviewed in [16]). The genetic interactions demonstrated that H antagonizes Notch signaling in a dose-dependent manner. Considering all the known interaction partners for CSL, H binds to Su(H) with the highest affinity (Kd = 2 nM) [4,17]. The Su(H)-interaction domain of H on its own is an unstructured random coil. After binding to the CTD of Su(H), H assumes a β-hairpin conformation (see Fig 2C and the manuscript in this issue [4]). Surprisingly, H interacts with specific side chains within the hydrophobic core of the Su(H)-CTD that are not exposed to the surface in the unbound structure of Su(H) [4]. The CTD of Su(H) is composed of a seven-stranded immunoglobulin (Ig)-fold (two β-sheets composed of three and four β-strands). This Ig-fold shows dramatic conformational changes when bound to H. H is sandwiched between the two β-sheets that compose the CTD, which is a hitherto completely new and unique interaction mode for Ig-folds. The conformational changes within the CTD block the CTD–NICD interaction and explain why binding of NICD and H are mutually exclusive. Based on their structural data, Yuan and colleagues designed specific point mutations within the CTD of Su(H), which lost H binding capacity but still was able to bind to NICD. In *D*. *melanogaster* in vivo experiments, using Notch-dependent wing and eye development as a readout, they could finally show that these Su(H) mutants have lost their corepressor activities but preserved their coactivator activity. These data highlight the importance of using *Drosophila* as a model system.

Considering the structure by Yuan et al. [4] in a broader context, the repressor structure also suggests that the on- and off-rates of the Su(H)/H corepressor complex are slow; this is in contrast to CSL/NICD/MAM coactivator. To date, a pulse of Notch signaling was mainly considered to be an interplay between receptor–ligand binding, posttranslational modifications of the NICD, and, ultimately, turnover of the coactivator complex [18]. Now, the rate of Su(H)-corepressors should be included in such considerations. Furthermore, the repressive mechanism at Notch target genes could also be a general theme used for other signaling pathways, like Wnt and Hedgehog signaling. For Hedgehog signaling, Gli is the central transcription factor, but the mechanisms of cofactor recruitment remain to be elucidated. For Wnt signaling, the central transcription factor is T cell factor (TCF)/Lymphoid enhancer binding factor (Lef), which in the absence of a Wnt signal binds promoters and recruits HDAC-containing corepressor complexes (reviewed in [19]).

## The CSL-Repressor Complex Configures Chromatin for the Notch Response

Regarding the repressive mechanism mediated by the CSL-repressor complex, H recruits Groucho and an HDAC-containing C-terminal binding protein (CtBP) corepressor complex [20–22]. The same is true for the human CSL-repressor complex containing HDACs and CtBP [23] (reviewed in [24]). Surprisingly, there is no direct Hairless homolog in mammals, but the functional homolog suggested by us and others is SHARP (also known as Spen or MINT). SHARP directly binds CSL, and intriguingly, it also interacts with the CTD of CSL similarly to Hairless [25].

Biochemical experiments from several laboratories implicated not only HDACs but also H3K4 demethylases as direct CSL-associated factors both in *Drosophila* [26–28] and mammals [28,29]. Recently, we added the counteracting H3K4 methyltransferase KMT2D as a novel component of the CSL coregulator complex [30]. All of these chromatin modifications are not only directed by a single transcription factor but most likely by a set of few transcription factors. The created balance between positive and negative histone marks sets the stage for the incoming extracellular signal.

The structure-based point mutants described in [4] gives us insights into how precisely mutagenesis can be used to dissect function of pivotal transcription complexes. Clearly, the next big step in the field is to solve the structure of human CSL/SHARP corepressor complex. Since CSL has been shown to function as a tumor suppressor [31], it might be feasible to design therapeutics that disrupt CSL-corepressor interactions in order to weakly activate Notch signaling, which may be beneficial in some disease settings.

Genome-wide studies using anti-CSL and anti-NICD antibodies have been important to define bona fide Notch target genes [32–34] in cells. Further analysis suggests that CSL occupancy depends on the presence of an active Notch signal [35,36], questioning the whole concept of CSL-bound corepressors. On the other hand, there are reports showing that deletion of CSL leads to derepression of some Notch target genes, both in *Drosophila* [20,37,38] and mammals [39]. Certainly, CSL knockout followed by rescue with wildtype or mutant CSL will be key to addressing this open question, leading the way forward to dissect individual functions of this central transcription factor. It will also be interesting to dissect the chromatin landscape at Notch target genes in the presence or absence of CSL or of individual corepressors.

In mammals, the situation of the CSL corepressor—namely SHARP [40,41], KyoT2 [42], and RITA [43] complex—is more complex, and the molecular mechanisms need to be further elucidated in the future. Clearly, as a next step, the cocrystal structures of CSL/SHARP and CSL/RITA would be a big move forward. (Fig 2D). This will unravel the molecular mechanisms whether or not the RAM-type or Hairless-type of binding to transcription factor CSL is the predominant one or if alternative types of interactions do exist.

## Abbreviations

ANK: ankyrin repeats
BTD: β-trefoil domain
CSL: CBF-1/RBP-J, Su(H), Lag-1
CtBP: C-terminal binding protein
CTD: C-terminal domain
HAT: histone acetyltransferase
HDAC: histone deacetylase
Ig: immunoglobulin
KDM: histone lysine demethylase
KMT: histone lysine methyltransferase
Lef: Lymphoid enhancer binding factor
MAM: Mastermind
NICD: Notch intracellular domain
NTD: N-terminal domain
PDB: protein data bank
RAM: RBP-J associated molecule
RBP-J: recognition binding protein of Jκ
RITA: RBPJ interacting and tubulin associated
SHARP: SMRT/HDAC1 associated repressor protein
Su(H): Suppressor of Hairless
TCF: T cell factor
